# Spatiotemporal constrained RNA–protein heterogeneous network for protein complex identification

**DOI:** 10.1093/bib/bbae280

**Published:** 2024-06-10

**Authors:** Zeqian Li, Shilong Wang, Hai Cui, Xiaoxia Liu, Yijia Zhang

**Affiliations:** School of Information Science and Technology, Dalian Maritime University, Dalian, 116026, China; School of Information Science and Technology, Dalian Maritime University, Dalian, 116026, China; School of Information Science and Technology, Dalian Maritime University, Dalian, 116026, China; Department of Neurology and Neurological Sciences, Stanford University, CA 94305, USA; School of Information Science and Technology, Dalian Maritime University, Dalian, 116026, China

**Keywords:** protein complex, spatiotemporal interaction pattern, RNA–protein interactions, heterogeneous network embedding

## Abstract

The identification of protein complexes from protein interaction networks is crucial in the understanding of protein function, cellular processes and disease mechanisms. Existing methods commonly rely on the assumption that protein interaction networks are highly reliable, yet in reality, there is considerable noise in the data. In addition, these methods fail to account for the regulatory roles of biomolecules during the formation of protein complexes, which is crucial for understanding the generation of protein interactions. To this end, we propose a **S**patio**T**emporal constrained **R**NA–protein heterogeneous network for **P**rotein **C**omplex **I**dentification (STRPCI). STRPCI first constructs a multiplex heterogeneous protein information network to capture deep semantic information by extracting spatiotemporal interaction patterns. Then, it utilizes a dual-view aggregator to aggregate heterogeneous neighbor information from different layers. Finally, through contrastive learning, STRPCI collaboratively optimizes the protein embedding representations under different spatiotemporal interaction patterns. Based on the protein embedding similarity, STRPCI reweights the protein interaction network and identifies protein complexes with core-attachment strategy. By considering the spatiotemporal constraints and biomolecular regulatory factors of protein interactions, STRPCI measures the tightness of interactions, thus mitigating the impact of noisy data on complex identification. Evaluation results on four real PPI networks demonstrate the effectiveness and strong biological significance of STRPCI. The source code implementation of STRPCI is available from https://github.com/LI-jasm/STRPCI.

## Introduction

Proteins are important for maintaining the structure, function and life activities of living cells. Due to their diversity and complexity, proteins play a crucial role in living systems [1]. Typically, proteins cannot independently participate in cellular activities but fulfill specific functions through collaborative interactions [2]. The rapid growth of protein–protein interaction (PPI) data through high-throughput technologies in recent years has provided a wealth of information for understanding protein action patterns and biological processes [3, 4]. Various protein interactions can be integrated into an interaction network in which some subregions form protein complexes. In this form, they affect cellular activities and participate in life processes.

Protein complex identification studies with computational methods have achieved remarkable success in recent years [5–7]. Based on the protein interaction network (PIN), protein complex identification can be converted to seek densely connected regions within the network. Existing methods can be divided into two categories. The first one merely focuses on PINs, which utilizes the topology structure in the PIN to identify protein complexes. Early on, some methods search for protein complexes by simple graph clustering [8, 9], but they ignore the fact that each protein can be involved in different protein complexes. In subsequent work, MCODE [10], one of the pioneers of network-based methods, weighs proteins according to local neighbor density, selects proteins with a high neighbor density as seed nodes and traverses outward to identify dense regions. Finally, regions with a certain density are retained as protein complexes. ClusterOne [11], which also measures density values, expands protein subsets by cohesiveness and merges overlapable groups. The CMC [12] is a clustering algorithm based on maximal cliques. It first obtains all the largest protein groups that can be formed in a PIN and then iteratively scores protein connections, identifies highly overlapping groups and optimizes them. The PEWCC [13] first performs a reliability metric on PPIs and filters a fraction of invalid connections. Weighted clustering coefficients are then used as protein clustering criteria to form denser and more accurate protein complexes. This line of researches highly depends on original noisy PPIs data, and ignores other valuable information for protein complex identification.

The second class of methods utilizes other available protein-related biological information as a complement to protein interaction information. COAN [14] uses gene ontology annotation-augmented networks on top of PIN to measure protein interactions jointly through network structural and functional annotation similarity. TSN-PCD [15] generates a series of sub-networks consisting of proteins active at the same time by integrating gene expression profiling data into PINs, and it carries out protein complex identification in these time-tagged sub-networks. Based on the core-attachment principal, CORE [16], COACH [17] and HUNTER [18] all first detect the highly coexpressed protein nodes contained in the PIN as the core and then add attachments according to some connectivity rules. Such methods are better able to express the structure of protein complexes. Apart from this, GANE [19] enhances PIN network information through GO attributes. Embedding similarity is used as a criterion for selecting cores and adding attachments. GHAE [6] integrates gene ontology attributes in heterogeneous networks, learns protein embeddings through attention mechanisms and screens core proteins and attachments based on embedding similarity. CO-DPC [20] screens active proteins based on gene expression profile information with the help of the 3-sigma principle and constructs dynamic PPI networks. CO-DPC identifies locally dense subgraphs as protein cores and further complements the attachments.

Although the second type of method is able to filter some of the false positives to a certain extent by introducing biological information, essentially, this information is about a certain property or expression of proteins, while the regulatory role of other types of biomolecules in the complex formation process is neglected. Inspired by the background knowledge that RNA acts in the assembly process of protein complexes [21, 22], we believe that it is necessary to introduce RNA as heterogeneous information into the PIN. In addition, several works have attempted to model the dynamic properties of proteins [23, 24], whereas most protein interactions and even protein complex formation occur in specific cellular compartments at specific cellular timescales. Thus, we expect to consider both temporal and spatial factors to ensure that protein associations in complexes are capable of occurring and are biologically meaningful.

Existing methods, while attempting to utilize the structural and other biological information of PINs, are limited by the expressive power of homogeneous PINs and the homogenization of the biological information to be learned [20, 25]. This further affects the biological significance of protein complex identification. Therefore, we try to fuse protein interaction information together with additional biological information into a heterogeneous network, thereby effectively integrating multi-source data and maintaining rich information.

Heterogeneous network representation learning [26] has shown strong modeling capabilities in recent years, and it aims to preserve the semantic information as well as both explicit and latent structural information conveyed by each type of node in a heterogeneous network. It maps these information to a low-dimensional vector representation of the target node. It is common to think that two nodes in a heterogeneous network are connected by a specific type of edge. However, as far as spatiotemporal information is concerned, protein nodes can be active in different temporal and spatial domains, so the network we try to construct becomes a complicated multiplex structure. Proteins exhibit different roles in different time-spaces, thus different spatiotemporal interactions between proteins have different contributions to their embeddings. How to effectively model the multiplex heterogeneity of the spatiotemporal constrained protein information network is the first problem we need to solve. In addition, instead of simply aggregating all the neighbor biological information to learn protein embeddings, how to model more advanced semantic information is another intractable issue. Protein nodes with more similar spatiotemporal expression patterns or similar roles in the complex are also more semantically and structurally consistent [27, 28]. Consideration of such indirect information is integral for obtaining high-quality protein embeddings.

To alleviate the above challenges, we propose a **S**patio**T**emporal constrained **R**NA-protein heterogeneous network for **P**rotein **C**omplex **I**dentification, namely STRPCI. STRPCI first integrates RNA information into PINs by constructing RNA–protein heterogeneous networks. In order to rationally integrate multiple spatiotemporal interaction behaviors into protein embedding, we define the concept of spatiotemporal interaction patterns to characterize the PPI substructure network under multiple spatiotemporal scenarios. To capture high-level structure information under multiple spatiotemporal conditions, we extract wide-domain interaction pattern and deep-domain interaction pattern. The protein embeddings in the multiplex heterogeneous protein information networks are then learned with a dual-view aggregator (DVA). Finally, contrastive learning is used to synergize the protein embedding information under the two spatiotemporal interaction patterns. STRPCI measures the tightness of protein interactions in terms of both spatiotemporal information constraints and biomolecular regulatory factors. Evaluation results on four real PINs show that STRPCI exhibits more advanced performance than other existing methods.

## Preliminaries

### Gene expression profile data

This paper uses saccharomyces cerevisiae gene expression profiles, which are obtained from the NCBI GEO database [29]. These data record the expression of saccharomyces cerevisiae in three consecutive metabolic cycles, where each metabolic cycle is subdivided into 12 expression times. We average the expression values of each gene over the three metabolic cycles to finally obtain their active expression values over the 12 time series.

### Subcellular localization data

This paper exploits subcellular localization data of saccharomyces cerevisiae, which are obtained from the COMPARTMENTS database [30]. Protein localization is important information that determines the function and activity of a protein in a cell to some extent. Different proteins may assume different biological functions in different parts of the cell. Specifically, there are 11 subcellular locations in the cell, which are extracellular space, nucleus, mitochondrion, endosome, vacuole, peroxisome, endoplasmic reticulum, golgi apparatus, cytosol, cytoskeleton and plasma membrane.

### Basic spatiotemporal interaction pattern

*Heterogeneous bioinformatic network*. We define a bioinformatic network as $G=\left \{V, E, X, A, R\right \}$, where $V$ is the set of bioinformatic nodes, and $E$ is the bioinformatic interactions. $X\in \mathbb{R}^{|V|\times h}$ denotes the original feature matrix of all the nodes in $V$, $n$ is the number of nodes and $h$ is the feature dimensionality. $A$ and $R$ are the set of all the bioinformatic node types and interaction types in $G$, respectively. Each bioinformatic node and interaction can be mapped to a specific node and interaction type; $G$ can be considered a heterogeneous bioinformatic network if $|A|+|R|>2$. Otherwise, it degenerates into a homogeneous network.

*Multiplex heterogeneous bioinformatic network*. $G$ is a multiplex heterogeneous bioinformatic network when the interactions are compounded between identical pairs of bioinformatic nodes.

*Basic interaction pattern*. In a multiplex heterogeneous bioinformatic network, we define the basic interaction pattern between two types of bioinformatic nodes $A_{i}$ and $A_{j}$ as $\mathcal{O}\{r_{1}, r_{2}, \dots , r_{|R|}\}$. $\mathcal{O}$ indicates that the interaction types in it are all optional. There is at least one kind of interaction form between $A_{i}$ and $A_{j}$. Based on this, our protein representation learning task in multiplex heterogeneous bioinformatic network can be viewed as learning the $d$-dimensional embedding vectors of protein nodes. Our goal is to make the final protein vectors draw on the heterogeneous information in the network, learn multiplex interaction relationships distinctly and capture high-level semantics.

*Basic spatiotemporal interaction pattern*. We take cellular time series and subcellular location as constraints on protein interactions, implying that the type of complex relationship between proteins is spatiotemporal information. Thus, we further develop the basic spatiotemporal interaction pattern with the concept of the basic interaction pattern. Based on the temporal range from $T_{1}$ to $T_{m}$ and the spatial range from $S_{1}$ to $S_{n}$ of protein interactions, the basic spatiotemporal interaction patterns that can be obtained include $m$ single time interaction patterns $\{T_{1}, \dots , T_{m}\}$, $n$ single spatial interaction patterns $\{S_{1},\dots ,S_{n}\}$ and $m\times n$ spatiotemporal overlap interaction patterns $\{T_{1}\&S_{1},\dots ,T_{m}\&S_{n}\}$.

## Method

**Figure 1:**
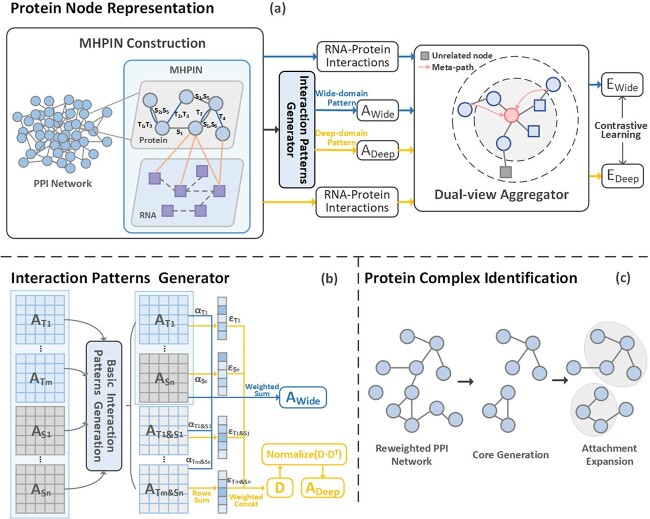
The overall structure of STRPCI. Part (a) is the framework for constructing MHPIN and the representation learning of protein nodes. It constrains the edges of the PPI network with spatiotemporal information and combines RNA–protein interactions to construct MHPIN. STRPCI extracts the wide-domain interaction pattern and the deep-domain interaction pattern, then learns different levels of neighbor structural information to enhance protein node representations with the DVA. Part (b) shows the specific process of wide-domain interaction pattern and deep-domain interaction pattern generation. Part (c) performs protein network reweighting based on the optimized protein node representations, mines core proteins and extends the attachment proteins based on node similarity to form complexes.

In this paper, a protein complex identification method based on spatiotemporal constrained RNA–protein heterogeneous network is proposed. Its main framework is shown in Fig. 1. In order to reasonably integrate the spatiotemporal expression information of proteins as well as the biological regulatory information, we construct a spatiotemporal constrained RNA–protein heterogeneous network. Then, we treat it as a Multiplex Heterogeneous Protein Information Network (MHPIN) to extract the wide-domain interaction pattern and deep-domain interaction pattern. We use a DVA to learn pattern embeddings and perform inter-pattern co-optimization through contrastive learning. Our method models protein pair affinities with spatiotemporal interaction patterns and further learns heterogeneous biological information. Finally, we employ the core-attachment strategy to identify protein complexes according to embedding distance reweighted PIN.

### MHPIN construction

First, we integrate RNA and RNA–protein interaction information as heterogeneous information into PIN. It is to model the regulatory role of RNA for protein interactions and strengthen the evidence of protein associations. According to the RNA–protein correlation coefficients in the RNAct database [31], we select for each protein node those that exceed the average value as its association node. For the constructed RNA–protein heterogeneous information network, the node set $V$ is the concatenation of the protein node set $V^{P}$ and the RNA node set $V^{R}$, i.e. $V = V^{P} \cup V^{R}.$

Further, we constrain protein interactions by time-course gene expression profile and subcellular localization data, parsing out those with co-active time or location. We introduce the time series and the cellular locations of interaction occurrences into the filtered PIN as the constraint information of the protein interactions. As shown in Fig. 1(a), it can actually be regarded as the type information of protein connecting edges.

Time-course gene expression profile data mark the active expression behavior from the cellular temporal perspective. It can reflect when the protein is involved in complex formation and achieves cellular function. Usually, global threshold is set to determine whether a protein is expressed [32]. However, this is not a fair strategy because the substantial expression levels of different proteins are distinct. We follow the practice of method [33] and calculate an independent gene expression threshold for each protein $p$ through the mean and standard deviation: 


(1) \begin{align*}& Active\underline{\ }threshold(p)=u_{p}+k \sigma_{p} \times\left(1-\frac{1}{1+\sigma_{p}^{2}}\right),\end{align*}

where $u_{p}$ is the mean value of protein activity and $\sigma _{p}$ is the standard deviation of protein activity. When $k$ is set to a larger value, the retained proteins are more certain to be active, but at the same time, the number of PPIs is reduced as a result, making the PINs excessively sparse. We tune the value of $k$ ranging from 0 to 3 and ultimately set it to 1 to balance the two aspects.

As shown in Fig. 1(a), we finally construct an MHPIN by integrating RNA information, protein co-expression information and co-location information.

The MHPIN is first decomposed according to the spatiotemporal interaction constraints to generate a series of temporal sub-networks and spatial sub-networks. We denote the adjacency matrix set of these sub-networks by $\{A_{r}\in R^{|V|\times |V|}|r=T_{1}, \ldots ,T_{m}, S_{1},\ldots , S_{n}\}$, where $|V|$ is the number of protein nodes, and $m$ and $n$ are the number of temporal and spatial sub-networks, respectively. According to time and location, basic spatiotemporal interaction patterns are generated for single time, single space and spatiotemporal overlap. If the protein interactions represented by the adjacency matrices occur in a particular temporal sub-network and a spatial sub-network simultaneously, they are recorded as $1$ in the corresponding basic spatiotemporal interaction pattern; otherwise, they are recorded as $0$. For example, if $p_{i}$ and $p_{j}$ are adjacent to each other in the $T_{1}$ sub-network and are adjacent to each other in the $S_{1}$ sub-network, the interaction values of $p_{i}$ with $p_{j}$ in the matrices of the basic spatiotemporal interaction patterns $T_{1}$, $S_{1}$ and $T_{1}\&S_{1}$ are all $1$. The presence of a zero matrix indicates that there is no protein interaction.

### Wide-domain interaction pattern

The wide-domain interaction pattern is a composite pattern that contains multiple basic spatiotemporal interaction patterns. It aims to adaptively learn the importance of basic spatiotemporal interaction patterns and to explain protein interaction patterns from the breadth of spatiotemporal scenarios, hence the name wide-domain interaction pattern.

After generating basic spatiotemporal interaction patterns, the wide-domain interaction pattern aggregator differentially learns the importance of each interaction pattern and maintains protein interactions with high spatiotemporal activity scenario similarity. Proteins with similar active patterns are embedded in closer proximity. Specifically, as the blue part of Fig. 1(b) shows, to capture multiplex spatiotemporal interactions between proteins, the wide-domain interaction pattern aggregator first aggregates each individual basic spatiotemporal interaction pattern: 


(2) \begin{align*}& {\tilde{\mathbf A}}_{Wd}=\sum_{i=1}^{M}\alpha_{i}{\bar{\mathbf A}}_{i},\end{align*}

where ${\tilde{\mathbf A}}_{Wd}$ denotes the adjacency matrix of basic spatiotemporal interaction patterns, and $\alpha $ is a vector of learnable weight parameters. The $M$ is the number of basic spatiotemporal interaction patterns.

We then take ${\tilde{\mathbf A}}_{Wd}$ as a protein–protein association matrix into the DVA module. The DVA consists of a first-order neighbor view and a meta-path view. In the wide-area interaction pattern aggregator, the information captured by the first-order neighbor view includes, on the one hand, information about proteins with which they have direct neighbor relationships. This part of the information also contains information about the similarity of the protein’s spatiotemporal interaction pattern. On the other hand, there are also direct RNA–protein associations, which capture information about RNAs that play a regulatory role in protein function. To capture deeper connections between basic spatiotemporal interaction patterns and co-regulated relationships between proteins directed by the same RNA, we perform bioinformatic aggregation in the meta-path view. Specifically, the aggregation of meta-path view is reflected in the fact that when proteins $q$ and $o$ have similar spatiotemporal interaction patterns to $p$ at the same time, $q$ and $o$ also show high similarity. In addition, protein interactions formed by the same RNA regulation are also crucial. It can complement some false-negative missing information.

DVA learns a structurally complementary protein embedding in each of the two views and then performs co-optimization between the two views, ultimately obtaining a wide-domain interaction pattern guided protein embedding $\mathbf{Z}^{wide}$.

### Deep-domain interaction pattern

The deep-domain interaction pattern is used to describe the local characteristics of protein nodes, including the types and quantities of basic spatiotemporal interaction patterns. It aims to explain the underlying associations between proteins by extracting structural attributes from the basic spatiotemporal interaction patterns, thereby justifying it is named as deep-domain interaction pattern.

The deep-domain interaction pattern portrays the role played by proteins in the complex from the perspective of spatiotemporal interaction behavior. Complexes usually contain several highly connected protein nodes with a significantly higher degree than average. These nodes usually exist as the core part in the complex and have more direct interactions with other proteins. Some peripheral nodes, on the other hand, exhibit low-degree characteristics. Based on this, the deep-domain interaction pattern is used to represent the spatiotemporal interaction density. It describes the interaction patterns of proteins in terms of their local interaction structure features. Intuitively, proteins that exhibit similar local structural features in a given scenario will tend to have more similar embedding representations.

The deep-domain interaction pattern aggregator takes a global view of aggregating features between proteins. As the yellow part shown in Fig. 1(b), firstly, a deep-domain interaction pattern matrix $\mathbf{D}$ is calculated from the individual spatiotemporal interaction patterns: 


(3) \begin{align*} &\begin{aligned} \mathbf{D}& =\varepsilon_{1}\cdot\mathbf{D}_{1}\parallel\varepsilon_{2}\cdot\mathbf{D}_{2}\parallel\cdots\parallel\varepsilon_{M}\cdot\mathbf{D}_{M} \\ &=(\mathbf{D}_{1}\parallel\cdots\parallel\mathbf{D}_{M})\cdot\boldsymbol{\Lambda}_{\varepsilon} \end{aligned} \end{align*}

(4) \begin{align*} & {\tilde{\mathbf A}}_{Dd}=normalize(\mathbf{D}\cdot\mathbf{D}^{\mathsf{T}}), \end{align*}

where $\varepsilon $ is a set of learnable weights, and $\mathbf{D}_{i(p)}=\sum _{q=1}^{|\mathbf{P}|}\bar{A}_{i(p,q)}$ is a column vector corresponding to the spatiotemporal interaction pattern $i$, which records the number of behaviors of all protein nodes associating with other proteins. The $\parallel $ is a concat operation, and $\mathbf{{\Lambda }}_{\varepsilon }$ is a learnable diagonal matrix consisting of all $\varepsilon $ vectors.

In the adjacency matrix ${\tilde{\mathbf A}}_{Dd}$ of deep-domain interaction pattern, the higher weights represent the closer roles played by the two protein nodes in each spatiotemporal scenario.

We use ${\tilde{\mathbf A}}_{Dd}$ as a protein–protein association matrix and feed it into the DVA module. The aggregation process in the DVA module is consistent with the corresponding part in the wide-domain interaction pattern aggregator. The difference is that for the target protein, in the deep domain interaction pattern aggregator, the first-order structure view captures the role information implied by the protein community structure. It is the deeper level of semantics. In the meta-path view, the deep-domain interaction pattern captures the indirect community structure similarity as well as the role correlation between proteins.

### Dual-view aggregator

The DVA module first aggregates first-order protein and RNA neighbors for the target protein node with an attention mechanism. Node-level attention can model the different contributions of neighbors of the same type: 


(5) \begin{align*}& \mathbf{z}_{p}^{\varphi}=\sigma\left(\sum_{q\in N_{p}^{\varphi}}\beta_{pq}\cdot \mathbf{H}_{q}\right),\end{align*}

where $\varphi $ denotes the type of the aggregated one-hop neighbor $q$, and $\mathbf{H}$ is the characteristic of the protein node, which is a row vector in $X$. The $\sigma $ is a nonlinear activation function. $\beta $ is the attention value of $q$ over $p$; it can be obtained in the following way: 


(6) \begin{align*}& \beta_{pq}=\frac{exp(LeakyReLU(b_{q}))}{\sum_{q\in N_{p}^{\varphi}}exp(LeakyReLU(b_{q}))},\end{align*}

where $b_{q} = \mu _{\varphi }^{\top }[h_{p}||h_{q}]$, || concatenates the features of proteins $p$ and $q$. The $\mu _{\varphi }$ stands for an attention vector on the node level.

We then use the following type-level attention on first-order neighbors to distinguish the importance of different types of biological information: 


(7) \begin{align*} & \mathbf{Z}_{p}^{stra}=\sum_{\varphi=1}^{K_{stra}}\gamma_{\varphi}\cdot \mathbf{z}_{p}^{\varphi} \end{align*}

(8) \begin{align*} & \gamma_{\varphi}=\frac{\exp\left(w_{\varphi}\right)}{\sum_{\varphi=1}^{K_{stra}}\exp\left(w_{\varphi}\right)} \end{align*}

(9) \begin{align*} & w_{\varphi}=\frac{1}{|N^{P}|}\sum_{i\in N^{P}}\mu_{stra}^{\top}\cdot\tanh\left(\mathbf{W}z_{p}^{\varphi}+\mathbf{b}\right), \end{align*}

where $K_{stra}$ is the type quantity of first-order neighbors. $\mu _{stra}$ is the attention vector in type level. $\mathbf{W}$ and $\mathbf{b}$ are two learnable parameters.

We model higher order interactions using meta-path form. Indirect relationships are represented by, for example, sequences such as protein–protein–protein sequences and protein–RNA–protein. Specifically, we first aggregate higher order protein neighbors under the same meta-path type: 


(10) \begin{align*}& \mathbf{z}_{p}^{\phi}=\frac{1}{d_{p}+1}\mathbf{h}_{p}+\sum_{q\in N_{p}^{\phi}}\frac{1}{\sqrt{(d_{p}+1)(d_{q}+1)}}\mathbf{h}_{q},\end{align*}

where $d_{p}$ and $d_{q}$ are, respectively, the degrees of proteins $p$ and $q$. The above formula is a meta-path specific graph convolutional network (GCN) [34]; we set it as a single layer in this work. The $\mathbf{z}_{p}^{\phi }$ is the embedding of protein $p$, which is guided to be learned by the meta-path $\phi $.

We then aggregate different types of meta-path information, which also have different contributions to protein embedding: 


(11) \begin{align*}& \mathbf{Z}_{p}^{mp}=\sum_{\phi=1}^{K_{mp}}\theta_{\phi}\cdot \mathbf{z}_{p}^{\phi},\end{align*}

where $K_{mp}$ is the type quantity of meta-path neighbors. $\theta $ can be acquired in the same way as $\gamma $ above.

Finally, the interaction pattern embeddings $\mathbf{Z}^{wide}$ and $\mathbf{Z}^{deep}$ are obtained by averaging the first-order neighbor embeddings $\mathbf{Z}^{stra}$ and metapath neighbor embeddings $\mathbf{Z}^{mp}$.

### Contrastive learning Objectives

STRPCI learns protein embeddings through a self-supervised approach. We define two contrastive learning objectives that model representation consistency between views and between interaction patterns by maximizing mutual information. These objectives, in turn, enhance the representational capability of our method.

First, contrastive learning is performed between the first-order neighbor view and the meta-path view in the DVA module. We take the same nodes in the first-order neighbor view and meta-path view as positive samples of each other and take other nodes in each other’s views as negative samples. Based on $\mathbf{Z}^{stra}$ and $\mathbf{Z}^{mp}$, our contrastive objective is defined as 


(12) \begin{align*}& \mathcal{L}_{stru}=-\sum_{p\in{V^{P}}}\log\frac{exp\left(s\left(\mathbf{Z}_{p}^{stra},\mathbf{Z}_{q}^{mp}\right)/\tau_{stru}\right)}{\sum_{q\in{V^{P}}} exp\left(s\left(\mathbf{Z}_{p}^{stra},\mathbf{Z}_{q}^{mp}\right)/\tau_{stru}\right)},\end{align*}

where $s(\cdot )$ is a function to measure the protein embedding distance, and we set it as the cosine similarity function. $\tau _{stru}$ is a temperature parameter.

In addition, we perform contrastive learning between the wide-domain interaction pattern and the deep-domain interaction pattern. The maximization of mutual information between these two allows the protein embedding to learn the information of different interaction patterns jointly. The contrastive loss is defined as follows: 


(13) \begin{align*}& \mathcal{L}_{pat}=-\sum_{p\in{V^{P}}}\log\frac{\exp(s(\mathbf{Z}_{p}^{wide},\mathbf{Z}_{q}^{deep})/\tau_{pat})}{\sum_{q\in{V_{P}}}\exp(s(\mathbf{Z}_{p}^{wide},\mathbf{Z}_{q}^{deep}) /\tau_{pat})},\end{align*}

where $\mathbf{Z}^{wide}$ and $\mathbf{Z}^{deep}$ are the node embeddings learned by the wide-domain interaction pattern aggregator and the deep-domain interaction pattern aggregator, respectively. $\tau _{pat}$ is a temperature parameter.

With the above two contrastive objectives, on the one hand, the structural data of MHPIN are reasonably and adequately considered. On the other hand, the deep information based on the basic spatiotemporal interaction patterns is fully explored and preserved. Finally, we jointly optimize the two contrastive loss functions by 


(14) \begin{align*}& \mathcal{L}=\mathcal{L}_{stra}+\mathcal{L}_{pat}\end{align*}

Finally, we perform average pooling of the wide-domain interaction pattern embeddings and the deep-domain interaction pattern embeddings to obtain the final protein representation: $\mathbf{Z}=1/2(\mathbf{Z}^{wide}+\mathbf{Z}^{deep})$.

### Embedding-based complex identification

Protein complexes usually consist of regions closely associated with the core function of a biological process and auxiliary proteins with greater dynamics. Biology defines this form as a core-attachment structure [27]. We use the core-attachment strategy for complex identification based on protein embeddings learned from STRPCI. As shown in Fig. 1(c), the protein complex identification process is generally divided into two stages: core generation and attachment expansion.

We first use protein embeddings to calculate the affinity between protein nodes and reweighted PINs by 


(15) \begin{align*}& \left.w_{pq}=\left\{\begin{array}{@{}ll}s(\mathbf{Z}_{p},\mathbf{Z}_{q}),&a_{pq}=1\\0,&a_{pq}=0\end{array}\right.\right.,\end{align*}

where $s(\cdot )$ is a cosine similarity function, and $\mathbf{Z}_{p}$ and $\mathbf{Z}_{q}$ are the embedding vectors of proteins $p$ and $q$, respectively. The value of $a_{pq}$ represents the existence of the adjacency in the original PIN.

In the first stage, we identify the core proteins based on a re-weighted PIN in the following steps:

(1) A clique mining algorithm is used to identify a set of clusters containing three or more proteins. The clusters are then checked for co-active times and co-active positions, and if the sets of these two are not all empty, then the clusters can be considered as a candidate core ($CCore$). All candidate cores are stored in a collection $Candidate\_Core$.

(2) Calculate the density score $d\_s$ of the candidate cores based on the protein embedding affinity and sort the candidate cores in descending order according to the $d\_s$ value: 


(16) \begin{align*}& d\_s(CCore_{i})=\sum_{p,q\in CCore_{i}}w_{pq}\end{align*}

(3) Based on the position in $Candidate\_Core$, the first candidate core with the largest $d\_s$ value $CCore_{1}$ is selected as a core and removed from $Candidate\_Core$. Then $CCore_{1}$ is put into a new set $Seed\_Core$.

(4) Iterate over the candidate cores in $Candidate\_Core$. If there is an overlap with $CCore_{1}$ and the number of non-overlapping nodes is less than 3, then delete such a candidate core. Otherwise, update the clusters of non-overlapping nodes into $Candidate\_Core$.

(5) Repeat the process from (2) to (4) until $Candidate\_Core$ is empty. At this time, the clusters in $Seed\_Core$ are all the eventually identified protein cores.

In the second stage, we extend the attachment proteins after identifying the core to form complete protein complexes. We calculate an adhesion score $ah\_s$ to measure the affinity between individual proteins and the cores: 


(17) \begin{align*}& ah\_s(p,Core_{j})=\frac{\sum_{q\in Core_{j}}w_{pq}}{|Core_{j}|},\end{align*}

where $p$ is not part of $Core_{j}$. Protein $p$ is considered to be able to join the complex structure as an attachment one when $ah\_s$ is greater than a threshold value $\lambda $. Finally, the core nodes and the attachment nodes form the identified complex together.

## Results and analysis

### Datasets and evaluation metrics

Our evaluations are conducted on three yeast PPI networks, which are Biogrid [35], Krogan14K [36] and Collins [37]. The details of these datasets are shown in Table 1, which records the number of proteins, the number of interactions and the average number of neighbors. The RNA–protein interaction data are downloaded from the RNAct database [31], which contains interaction data between 5963 RNAs and 7029 proteins. The gold standard protein complexes used as comparisons are from MIPS [38], CYC2008 [39], SGD [40], Aloy [41] and TAP06 [27], totaling 789.

**Table 1:** The PPI datasets used in the evaluations

**PPI networks**	**Number of** **proteins**	**Number of interactions**	**Average number of neighbors**
Biogrid	5640	59 748	21.187
Krogan 14K	3581	14 076	7.861
Collins	1622	9074	11.189
HPRD	7307	29 213	7.996

We calculate the matching degree between the identified protein complexes and the gold standard protein complexes with the affinity score formula. For one of the complex $p$ in the set of identified protein complexes $P$, and one of the complex $b$ in the set of gold-standard protein complexes $B$, we perform the following calculation: 


(18) \begin{align*}& NA(p,b)=\frac{|V_{p}\cap V_{b}|^{2}}{|V_{p}|\times|V_{b}|},\end{align*}

where $V_{p}$ and $V_{p}$ denote the set of proteins in $p$ and $b$, respectively. The $|\cdot |$ denotes the number of proteins. Following previous work, two protein complexes are considered to be matched when the NA score exceeds 0.25.

From the perspective of matching score NA of protein complexes, this corresponds to three main metrics: Precision, Recall and F-score.

Precision is the proportion of identified complexes that can match at least one gold standard complex: 


(19) \begin{align*} & N_{cp}=|\{p\mid p\in P,\exists b\in B,NA(p,b)\geq\omega\}| \end{align*}

(20) \begin{align*} & Precision=\frac{N_{cp}}{|P|} \end{align*}

Recall is the proportion of gold standard complexes that can match at least one identified complex: 


(21) \begin{align*} & N_{cb}=|\{b\mid b\in B,\exists p\in P,NA(p,b)\geq\omega\}| \end{align*}

(22) \begin{align*} & Recall=\frac{N_{cb}}{|B|} \end{align*}

as mentioned earlier, the value of the threshold $\omega $ is usually set to 0.25. The F-score is the harmonic mean of Precision and Recall, so it exists as a composite criterion and is calculated as follows: 


(23) \begin{align*}& F\text{-score}=\frac{2\times\text{Precision}\times\text{Recall}}{\text{Precision}+\text{Recall}}\end{align*}

In addition, Accuracy evaluates protein complexes in terms of clustered sensitivity (Sn) and clustered positive predictive value (PPV), which are calculated as follows: 


(24) \begin{align*} &Acc=\sqrt{Sn\cdot PPV} \end{align*}

(25) \begin{align*} &Sn=\frac{\sum_{b=1}^{|B|}\max_{p=1}^{|P|}\left\{T_{bp}\right\}}{\sum_{b=1}^{|B|}N_{b}} \end{align*}

(26) \begin{align*} &PPV=\frac{\sum_{p=1}^{|P|}\max_{b=1}^{|B|}\left\{T_{bp}\right\}}{\sum_{p=1}^{|P|}\sum_{b=1}^{|B|}T_{bp}}, \end{align*}

where $T_{bp}$ is the number of proteins present in both $p$ and $b$, and $N_{b}$ is the number of proteins in the gold standard complex $b$.

### Performance comparison

**Figure 2:**
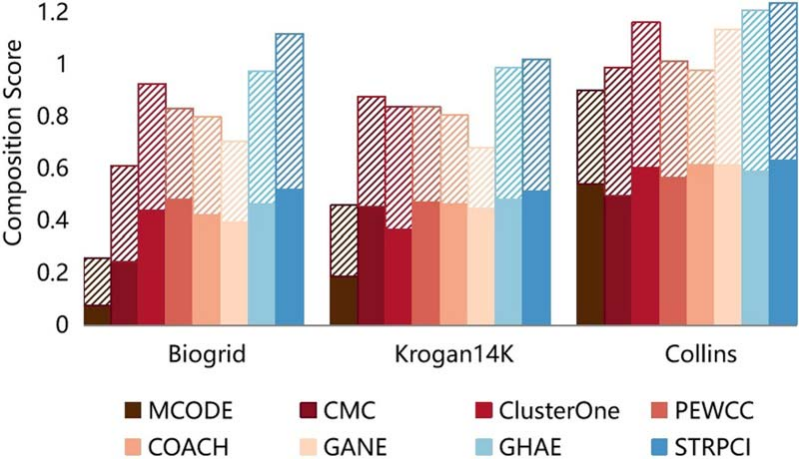
The performance of the complex identification methods on the composition score of F-score and Acc. The solid areas are the F-scores and the slashed areas are the Acc scores.

In this section, we analyze STRPCI in comparison with seven competitive methods, namely MCODE [10], CMC [12], ClusterONE [11], PEWCC [13], COACH [17], GANE [19] and GHAE [6]. Among these baselines, the first four are PPI network-based methods only, and the last three add additional biological information about the proteins. The protein complex identification performance on the three yeast datasets is shown in Table 2; the top-performing methods and the highest scores under various evaluation metrics have been highlighted in bold. To further visualize the performance on the two overarching performance metrics, F-score and Accuracy, we have also compared the combination scores in Fig. 2.

**Table 2:** Performance comparison of identified protein complexes on four benchmark PPI networks

**Species**	**Datasets**	**Methods**	**Precision**	**Recall**	**F-score**	**Acc**
Yeast	Biogrid	MCODE	0.276	0.043	0.075	0.181
		CMC	0.157	0.553	0.245	0.365
		ClusterOne	0.393	0.497	0.439	0.486
		PEWCC	0.375	0.677	0.483	0.347
		COACH	0.311	0.657	0.422	0.376
		GANE	0.345	0.464	0.395	0.310
		GHAE	0.443	0.489	0.465	0.509
		**STRPCI**	0.492	0.550	**0.520**	**0.598**
Yeast	Krogan 14K	MCODE	0.612	0.112	0.189	0.270
		CMC	0.472	0.440	0.455	0.421
		ClusterOne	0.467	0.302	0.366	0.473
		PEWCC	0.535	0.418	0.470	0.367
		COACH	0.461	0.465	0.463	0.342
		GANE	0.448	0.442	0.445	0.234
		GHAE	0.537	0.437	0.482	0.505
		**STRPCI**	0.593	0.450	**0.512**	**0.509**
Yeast	Collins	MCODE	0.847	0.400	0.540	0.362
		CMC	0.680	0.390	0.496	0.493
		ClusterOne	0.733	0.511	0.602	0.561
		PEWCC	0.837	0.426	0.564	0.449
		COACH	0.749	0.522	0.615	0.362
		GANE	0.819	0.491	0.615	0.520
		GHAE	0.609	0.571	0.590	**0.619**
		**STRPCI**	0.716	0.563	**0.631**	0.605
Human	HPRD	MCODE	0.206	0.026	0.046	0.162
		CMC	0.364	0.018	0.034	0.099
		ClusterOne	0.231	0.172	0.197	0.232
		PEWCC	0.274	0.230	0.250	0.226
		COACH	0.247	0.389	0.302	0.356
		GANE	0.484	0.575	0.526	0.511
		GHAE	0.476	0.654	0.551	0.579
		**STRPCI**	0.497	0.648	**0.563**	**0.595**

Overall, it can be seen in Fig. 2 that STRPCI achieves the optimal combination score on all three yeast datasets. This indicates that STRPCI can better balance Precision and Recall and effectively identify protein complexes. It should be noticed that STRPCI is not as good as GHAE in Accuracy on the Collins dataset, and we conclude the reason is that GHAE identifies more protein complexes with high match levels, but our method is superior in identification precision, focusing on complexes at both medium and high match levels. Also, our method is still superior to GHAE in the overall measure of F-score and composition score.

In addition, the precision performance of all baseline methods on the Biogrid dataset is significantly decreased, especially those focusing only on the PPI network. This is due to the fact that the Biogrid dataset has the highest average number of neighbors and will be more susceptible to the structure of the original PPI network as well as the false positive samples. GHAE is relatively robust due to the fact that it uses a heterogeneous network for the effective integration of GO information. STRPCI also achieves strong robust performance because it focuses on deep interaction patterns and bioregulatory information. The effective modeling of additional biological information increases the confidence of the PPIs, thus guaranteeing precision in the identification of the complexes.

To validate the effectiveness of the STRPCI method for the identification of human protein complexes, we also conduct performance evaluations and comparisons on the human dataset HPRD. The gold standard protein complexes are obtained from the HPRD database, totaling 1514. Notably, due to the lack of suitable time-course gene expression data for humans, we only utilize subcellular localization information of proteins as the basic spatial interaction pattern, from which we further extract the wide-domain and deep-domain interaction patterns.

As shown in Table 2, STRPCI achieves the best F-score and Acc compared with the other seven baseline methods on the HPRD dataset. Despite the lack of time-course gene expression data to provide more precise constraints on interaction conditions, STRPCI still performs well. This fully demonstrates that our method can effectively model complicated heterogeneous information and is well suited for application to human PPI networks.

### Ablation study

In order to investigate the effectiveness of the two interaction patterns in our model and the DVA, we design the following variant models for validation:

-w/o Pat: We do not consider protein spatiotemporal interaction patterns in this variant model. PPIs without co-active times or co-interaction locations are removed from the PIN and then constructed as heterogeneous networks along with RNA information for subsequent representation learning. Correspondingly, inter-pattern contrast is not present in this variant.

-w/o Wide: In this variant, we remove wide-domain interaction patterns, which means that only deep-domain spatiotemporal interaction patterns are retained to perform dual-view neighbor aggregation.

-w/o Deep: In this variant, we ignore deep-domain interaction patterns, which means that only wide-domain spatiotemporal interaction patterns are retained to perform dual-view neighbor aggregation.

-w/o Dva: In this variant, we replace the DVA with a two-layer GCN to aggregate direct and indirect neighbor node features.

-w/o RNA: In this variant, we construct protein multiplex spatiotemporal interaction networks and no longer model the regulatory role of RNA for proteins.

The ablation results are listed in Table 3; the top-performing methods and the highest scores under various evaluation metrics have been highlighted in bold. From the results, we observe that (1) the w/o Pat variant shows a significant decrease in accuracy compared with STRPCI. This suggests that protein spatiotemporal interaction patterns can, to some extent, capture deep spatiotemporal interaction information and strengthen the accuracy of protein association judgment. (2) Removing any of the spatiotemporal interaction patterns in the deep or wide domain decreases the F-score, but is slightly better compared with w/o Pat. Thus, they successfully play a positive role in different pattern learning, respectively. (3) Compared with GCN, the DVA can model the differential roles of different neighbors more efficiently and learn information about different structures more clearly and explicitly under the division of two views. (4) The performance of w/o RNA is lower than that of STRPCI, suggesting that RNA information, as an intermediate node for some protein interactions, can effectively model its regulatory role for protein interactions and even protein complexes.

**Table 3:** Performance of protein complex identification of STRPCI and its variants

**Datasets**	**Methods**	**Precision**	**Recall**	**F-score**	**Acc**
Biogrid	w/o Pat	0.396	0.632	0.487	0.454
	w/o Wide	0.432	0.567	0.490	0.533
	w/o Deep	0.411	0.624	0.495	0.497
	w/o Dva	0.421	0.511	0.462	0.537
	w/o RNA	0.444	0.584	0.504	0.558
	**STRPCI**	0.492	0.550	**0.520**	**0.598**
Krogan 14K	w/o Pat	0.571	0.407	0.475	0.427
	w/o Wide	0.576	0.428	0.491	0.477
	w/o Deep	0.592	0.425	0.495	0.493
	w/o Dva	0.603	0.398	0.480	0.422
	w/o RNA	0.587	0.442	0.504	0.498
	**STRPCI**	0.593	0.450	**0.512**	**0.509**
Collins	w/o Pat	0.678	0.489	0.568	0.492
	w/o Wide	0.688	0.508	0.584	0.499
	w/o Deep	0.707	0.506	0.590	0.463
	w/o Dva	0.668	0.485	0.562	0.505
	w/o RNA	0.712	0.520	0.601	0.552
	**STRPCI**	0.716	0.563	**0.631**	**0.605**

### Parameter sensitivity analysis

**Figure 3:**
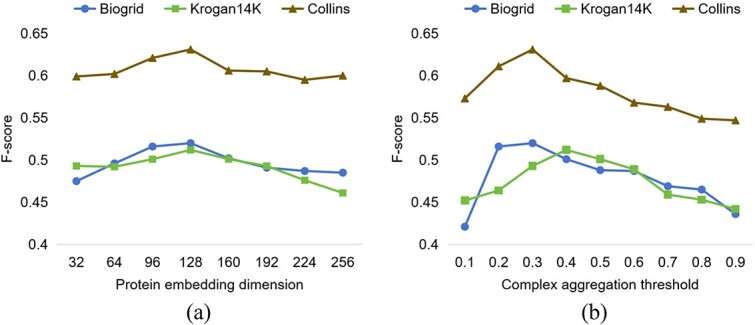
Sensitivity of the embedding dimension $d$ and aggregation threshold $\theta $.

We evaluate the sensitivity of STRPCI to two main parameters, including the protein embedding dimension, and the attachment aggregation threshold. The F-scores on the three yeast datasets are given in Fig. 3.

#### Effect of protein embedding dimension $d$

We test the protein embedding dimensions in the range from 32 to 256. As shown in Fig. 3(a), the F-score achieves the highest performance in 128 dimensions on all three yeast datasets. It indicates that 128 dimensions is the most suitable choice for protein node embedding in our method. In addition, the performance is at a lower state when the dimension is near 32. This is due to the fact that the compression for the embedding dimension leads to an inadequate representation of complicated information. It also starts to stabilize at lower levels at 192 to 256 dimensions, which is due to the fact that the model begins to focus on unimportant information.

#### Effect of complex aggregation threshold $\lambda $

The complex aggregation threshold determines what kind of proteins can be further assembled as attachment proteins after identifying the protein cores. We observe from Fig. 3(b) that the F-scores of different $\lambda $ values among the three datasets show the same tendency to increase and then decrease. When the threshold is too low, it rapidly increases the number of attached proteins and subsequently elevates the ratio of irrelevant proteins. On the contrary, when the threshold gets too large, it will filter a part of the effective proteins.

### Case study of identified complexes

In this section, we provide some of the protein complexes we identified that do not match the gold standard protein complexes by NA score indication. To further understand the biological significance of these protein complexes, we perform functional enrichment analyses to evaluate their functional relevance. The specific evaluation metric is the $P$-value [42] given by the LAGO tool [43], which is calculated as follows: 


(27) \begin{align*}& P\text{-value}=1-\sum_{i=0}^{k-1}\frac{{\left(\begin{array}{c}{|F|}\\{i}\end{array}\right)}{\left(\begin{array}{c}{|V|-|F|}\\{|p|-i}\end{array}\right)}}{\left(\begin{array}{c} {|V|}\\{|p|}\end{array}\right)},\end{align*}

where an identification complex $p$ contains k proteins of functional group $F$, and $|V|$ is the total number of proteins contained in the PPI network. Typically, the $P$-value has a cutoff value of 0.01, with values less than 0.01 considered biologically significant, while values greater than 0.01 are considered functionally irrelevant [44].

Table 4 lists the mismatched complexes identified by our model, and we can observe that their $P$-values are significantly less than 0.01, so they are biologically plausible potential complexes.

**Table 4:** Examples of mismatched identification protein complexes

**ID**	**Protein complex**	**Size**	**Min P-value**	**GO-Description**
1	YAL027W YLR135W YML095C YMR201C YOL090W	5	6.83018e-13	removal of nonhomologous ends
2	YML062C YDL229W YLL039C YER151C YDR381W YDR138W YJL168C	7	5.49518e-07	transcription-coupled nucleotide-excision repair
3	YDL139C YGL086W YGL116W YGL229C YGR014W YJL013C YNL236W YOR026W	8	3.17475e-09	mitotic cell cycle process
4	YBR010W YBR112C YCR084C YDL126C YDR510W YGR083C YNL021W YNL167C YOL004W	9	2.31864e-08	transcription corepressor activity
5	YDL132W YDR054C YDR099W YDR328C YFR004W YJR090C YLL050C YLR429W YOL133W YOR080W	10	3.01701e-14	SCF-dependent proteasomal ubiquitin -dependent protein catabolic process
6	YGL001C YGL012W YGR060W YGR175C YHR007C YHR072W YHR190W YLR056W YLR100W YML008C YML012W YMR015C YNL280C	13	4.82486e-29	cellular lipid biosynthetic process

In addition, we compare the matched but not identical protein complexes with the gold standard protein complexes. In Table 5, bold fonts are used to indicate the overlapping proteins with standard complexes among the identified complexes. The gold standard protein complex 1 in Table 5 is the conserved glucose-induced degradation (GID) complex. The complex 1 identified by the STRPCI method has a redundant node YDL176W. There have been in total 11 indications of the association of this redundant node with the GID complex [45], some of which are confirmed by pull-down or genetic interaction experiments, and these pieces of evidence indicate that YDL176W is strongly linked to the GID complex. For the gold standard complex 2, our identified complex in the Krogan14K dataset is consistent with its size. The only mismatched node, YDL165W, is not included in the Krogan14K dataset, while our identified YMR099C is highly similar to the other nodes in terms of their spatiotemporal action. For the gold standard complex 3, which contains seven protein nodes that do not interact in exactly the same cellular time-series and sub-locations, it can be found that the matching degree between the identified complexes and the gold standard complex 3 is still higher than the other two methods, which attributes to the efficient modeling of the biological regulatory information. On the whole, both GHAE and PEWCC methods identified protein complexes with lower matches than STRPCI, and the mismatched proteins have no relevant biological significance. This suggests that our methods can effectively model spatiotemporal expression information and biological regulatory information.

**Table 5:** Examples of matched but not identical protein complexes

**Method**	**Protein complex**	**Size**	**Mismatched number**
Gold standard 1	**YDR255C YCL039W YIL017C YGL227W YBR105C YIL097W YMR135C**	7	−
PEWCC	**YDR255C YIL017C YBR105C YIL097W** YKR059W YBL080C	6	3
GHAE	**YDR255C YCL039W YIL017C YGL227W YBR105C YMR135C** YBR198C YDR252W	8	2
STRPCI	**YDR255C YCL039W YIL017C YGL227W YBR105C YIL097W YMR135C** YDL176W	8	1
Gold standard 2	**YER068W YPR072W YIL038C YCR093W YDL165W**	5	−
PEWCC	**YER068W YPR072W YCR093W** YCR009C YDR519W	5	2
GHAE	**YER068W YPR072W YIL038C YCR093W** YLR330W YJL203W YDR502C	7	3
STRPCI	**YER068W YPR072W YIL038C YCR093W** YMR099C	5	1
Gold standard 3	**YDL084W YDR138W YML062C YHR167W YNL139C YNL253W YDR381W**	7	−
PEWCC	**YDL084W YML062C YHR167W YNL139C** YEL026W	5	3
GHAE	**YDL084W YDR138W YML062C YHR167W YDR381W** YGR030C YER164W	7	2
STRPCI	**YDL084W YDR138W YML062C YHR167W YNL139C YDR381W**	6	1

## Conclusion

In this paper, we propose a spatiotemporal constrained RNA–protein heterogeneous network for protein complex identification, STRPCI. We first construct an MHPIN with gene expression profiles, subcellular localization and RNA–protein interaction data. Then, wide-domain interaction patterns and deep-domain interaction patterns are extracted to capture deeper semantic information, and the information of direct neighbors and meta-path guided neighbors are aggregated with a dual-view neighbor aggregator. Finally, we jointly optimize the two pattern embeddings by contrastive learning. The PPI network is reweighted after obtaining protein embeddings, and protein complexes are identified with the core-attachment strategy.

## Future work

While STRPCI has achieved competitive results, there remain limitations and opportunities for improvement. Firstly, due to the lack of spatiotemporal data on RNA–protein interactions, we have not yet delved into the spatiotemporal regulation of RNA on protein complexes. In the future, with a more comprehensive dataset, we expect to better constrain and explore the factors that affect PPIs. Secondly, leveraging hard-links derived from biological experiments to partition data during the learning and validation stages would further enhance the performance and biological relevance of protein complex identification. Finally, we need to proceed with the exploration of methods that are fully applicable to the identification of human protein complexes on the basis of the available data.

**Key Points**

- Our work highly integrates multi-source biological information by constructing MHPINs, and the rational network structure is the primary condition for mining complicated information.
- We propose STRPCI, which achieves a deep comprehension of the spatiotemporal conditions of protein interactions by extracting spatiotemporal interaction patterns. STRPCI uses a DVA to learn direct and indirect neighbor information, effectively modeling the regulatory role of RNAs for protein interactions and even for the process of complex formation.
- Evaluation results on four datasets show that STRPCI achieves superior performance. In addition, the discussion of mismatched complexes and matched but not identical complexes suggests that STRPCI has strong biological significance.

## Acknowledgment

This work is supported by grant from the Natural Science Foundation of China (No. 62106034).

## Data availability

Data will be made available on request.

## Author Biographies

**Zeqian Li** received a bachelors degree from Taiyuan Normal University in 2020. She is studying for a masters degree in Computer Science and Technology Department of Dalian Maritime University. Her research interests include bioinformatics and data mining.

**Shilong Wang** received a bachelors degree from Dalian Maritime University in 2022. He is studying for a masters degree in Computer Science and Technology Department of Dalian Maritime University. His research interests include natural language processing and bioinformatics.

**Hai Cui** received the PhD degree from the College of Computer Science and Technology, Jilin University, China, in 2023. He is currently a post doctor in College of Information Science and Technology, Dalian Maritime University, China. His research interests include biomedical knowledge graph, drug repurposing and drug–disease association prediction.

**Xiaoxia Liu** is currently a post doctor, Department of Neurology and Neurological Sciences, Stanford University. Her main research interests include bioinformatics and text mining.

**Yijia Zhang** received the BSc, MSc and PhD degrees from the Dalian University of Technology, China, in 2003, 2009 and 2014. He is a professor in the School of Information Science and Technology at the Dalian Maritime University. He has published over 60 research papers on bioinformatics and text mining. Draft manuscript prepared by the author.
